# Trajectory of psychological distress and influencing factors in patients with gynecological cancers: a longitudinal study

**DOI:** 10.3389/fonc.2024.1380916

**Published:** 2024-11-22

**Authors:** Feifei Li, Jing Xu, Yueyuan He, Shanhong Zhang

**Affiliations:** Department of Nursing, The Second Hospital of Dalian Medical University, Dalian, China

**Keywords:** gynecological cancer, psychological distress, trajectory, potential category growth model, longitudinal study

## Abstract

**Objective:**

To explore the dynamic changes of psychological distress among patients with gynecological cancers across their treatment journey and identify the characteristics of subgroups of patients with distinct trajectories.

**Methods:**

This study included a convenience sample of 132 patients with cervical cancer, ovarian cancer or endometrial cancer who received surgery and adjuvant chemotherapy in the gynecological department of a Grade III and Class A general hospital in Liaoning Province between November 2022 and October 2023. Patients’ mean age was 55.46 ± 11.12 years. Psychological distress was measured with the Distress Thermometer and Problem List 1 day before surgery, before the first chemotherapy session, at the third chemotherapy session, and at the end of the last chemotherapy session. A latent class growth model (LCGM) was used to identify trajectories of psychological distress and explore influencing factors.

**Results:**

The LCGM identified three different trajectories of psychological distress in patients with gynecological cancers, including Class 1, “high level decline “ (37.4%), Class 2, “no psychological distress” (24.1%), and Class 3, “high level stable” (38.5%). Multinomial logistic regression analysis showed monthly per capita household income, disease type, positive coping style and temperament type were significant predictors of trajectory of psychological distress in patients with gynecological cancers.

**Conclusions:**

This study showed heterogeneity in the trajectory of psychological distress among patients with gynecological cancers. These findings should raise awareness among healthcare providers of the need to implement screening measures and timely psychological interventions in this patient population.

## Introduction

Globally, gynecological cancers are among the most common malignancies in women, with an estimated 3.6 million new cases of gynecological cancers and 1.3 million deaths from these cancers each year (1). In China, gynecological cancers account for 18.6% of all cancer incidence and 15.3% of all cancer-related deaths in women, with an annual incidence of 210,000 and mortality of 70,000-80,000. The main types of gynecological cancers are ovarian cancer, cervical cancer and endometrial cancer (2).

The psychological well-being of 30%-40% of patients with cancer is affected by their diagnosis and associated treatment (3–5). The National Comprehensive Cancer Network (NCCN) recognizes cancer-related psychological distress as unpleasant psychological (i.e., cognitive, behavioral, emotional), social, spiritual and/or physical reactions to a cancer diagnosis and the treatment process (6). Psychological distress in patients with cancer can impact quality of life, treatment compliance and survival (3–5). The occurrence of psychological distress in patients with cancer has been related to several factors, including temperament type, coping style and social support (7–11).

Psychological distress in patients with cancer has been conceptualized (12). Antecedents of psychological distress include treatment complexity. Attributes constitute depression, anxiety, coming to terms with living with a life-limiting disease, loss of hope and a perceived inability to cope. Consequences of these attributes can be positive, with patients focusing on living in the present, but are usually negative, ranging from mild mood disturbances to loss of coping strategies, with family and friends affected (12). Risk factors for psychological distress in patients with cancer include sex, age, employment and education level, cancer type and stage, emotional and physical comorbidities and treatment setting (inpatient vs outpatient) (13).

Due to advances in medical science, the survival of patients with gynecological cancers in China has improved, with 5-year relative survival rates of 55.9% for ovarian cancer, 75.9% for cervical cancer, and 87.7% for endometrial cancer (14). As patients with cancer are living longer, there is a need to prevent cancer-related psychological distress and manage its impacts with appropriate care and treatment. For patients with life-limiting conditions, the prevention, diagnosis and treatment of psychological distress may optimize end-of-life care.

At present, the trajectory of psychological distress has been investigated in patients with various cancers, including breast cancer and esophageal cancer (15, 33). Current research on psychological distress in patients with gynecological cancers is focused on cross-sectional studies of patients with cervical cancer or ovarian cancer. However, the psychological state of patients diagnosed with cancer is constantly changing from diagnosis through hospital admission, surgery and postoperative adjuvant therapy.

The objective of this longitudinal study was to explore the dynamic changes in psychological distress among patients with gynecological cancers across their treatment journey. Trajectories were modeled using a latent class growth model (LCGM), and the characteristics of subgroups of patients with distinct trajectories were identified (16). The LCGM is a statistical technique that allows for the identification of unobserved subgroups (or classes) within a population based on longitudinal data. Unlike traditional longitudinal models that assume a homogeneous population with a single trajectory, the LCGM acknowledges the existence of heterogeneity within the data and identifies distinct patterns of change over time. The primary advantage of LCGM is its ability to classify patients into different trajectories based on these changes, allowing for a nuanced understanding of patient experiences. The approach is especially valuable in psychological distress research in patients with cancer, where individual responses to cancer diagnosis and treatment may vary significantly. In this study, the model analyzed multiple measurements of psychological distress (recorded at four time points: pre-surgery, pre-first chemotherapy, during the third chemotherapy session, and post-treatment) to describe how distress levels evolve throughout the treatment process. According to stress theory and in accordance with previous literature (7–11), coping style, temperament type and social support were investigated as potential factors influencing psychological distress, as evidence suggests these variables may have a significant effect on psychological distress.

## Methods

### Study population

This study included a convenience sample of patients with cervical cancer, ovarian cancer or endometrial cancer who received surgery and adjuvant chemotherapy in the gynecological department of a Grade III and Class A general hospital in Liaoning Province between November 2022 and October 2023. Inclusion criteria were: 1) aged ≥18 years; 2) diagnosis of cervical cancer, endometrial cancer or newly diagnosed ovarian cancer on biopsy before surgery; 3) receiving initial surgery and postoperative chemotherapy; 4) aware of their disease; and 5) gave voluntary informed consent to participate in the study. Exclusion criteria were: 1) non-ovarian cancer diagnosed on pathology during surgery; 2) not recommended for adjuvant chemotherapy; 3) local or distant metastasis occurred during follow-up; 4) presence of other serious systemic diseases; 5) presence of a consciousness disorder or cognitive dysfunction; 6) history of mental disorders; or 7) loss to follow up, defined as failure to answer three consecutive phone calls at one follow-up timepoint.

This study was approved by the Ethics Committee of the participating hospital (Approval number: HLZQ20221104), and all patients provided signed informed consent.

### Data collection

Data collection was guided by a literature review that revealed the factors that commonly influence psychological distress and how psychological distress is measured in patients with cancer.

### Patient characteristics

Patient socio-demographic and clinical characteristics that were expected to impact psychological distress were collected using a questionnaire. Socio-demographic characteristics included age, place of residence, educational level, payment method for healthcare, monthly per capita household income, marital status, occupational status, and parity. Clinical characteristics included diagnosis and stage of disease, duration of disease, and comorbidities.

### Psychological distress

Psychological distress was measured with the Distress Thermometer and Problem List (DT&PL) (17). The DT was developed to screen for symptoms of distress. The DT is a single-item tool that measures a patient’s distress over the last week on a 0 (no distress) to 10 (extreme distress)–point Likert scale. The PL contains 5 scales and 40 items relevant to practical problems (6 items), communication problems (4 items), emotional problems (9 items), physical problems (20 items), and religious beliefs (1 item). The DT&PL have been translated into Chinese and the DT has been validated in a sample of 574 cancer patients in Beijing Cancer Hospital, showing good retest reliability (r=0.77, P < 0.01.) (18).

### Coping strategies

Coping strategies were measured with the Simplified Coping Style Questionnaire (SCSQ) (19). The SCSQ contains 20 items in two dimensions: positive coping styles (12 items) and negative coping styles (8 items). The SCSQ was validated in a sample of 20 Chinese university students with high Cronbach’s α coefficient (0.9) (19).

Perceived Social Support Scale Patient perceived social support was measured with the Perceived Social Support Scale (PSSS). The scale consists of 12 items and 3 dimensions: family support, friend support and other support. The PSSS is scored on a 7-point Likert scale ranging from 1 “totally disagree” to 7”strongly agree”, with higher scores or total scores on each dimension indicating a greater ability to perceive social support (20, 21). The Cronbach’s α coefficient of the scale is 0.85.

The Chinese version of the Eysenck Personality Questionnaire Revised Short Scale Personality traits were measured with the Chinese version of the Eysenck Personality Questionnaire Revised Short Scale (EPQ-RSC) (22). This scale consists of 48 items answered ‘yes’ or ‘no’, with corresponding scores identifying personality type as neuroticism (phlegmatic or melancholic) or extroversion (sanguine or choleric).

The scale has good reliability and validity, with a Cronbach’s α coefficient of 0.825, and has been widely used in China.

### Study design

This was a longitudinal study. Data were collected at 4 time points: 1 day before surgery (T1), before the first chemotherapy session (T2), at the third chemotherapy session (T3), and at the end of the last chemotherapy session (T4). At T1, the DT&PL, SCSQ, PSSS and EPQ-RSC were completed during interviews with patients. At T2-T4, the DT&PL was completed during follow-up by telephone, WeChat or in the outpatient department.

All data were recorded in a database and checked for accuracy.

### Statistical analysis

Data management and statistical analysis were performed with SPSS25.0 and Mplus8.0. Categorical data are reported as frequencies and were compared with the χ^2^ test. Normally or non-normally distributed continuous data are reported as mean ± standard deviation or median and interquartile range, respectively. Continuous data were compared by one-way analysis of variance. LCGM analysis was conducted with Mplus8.0. Models with increasing numbers of classes were estimated and model fit was compared. The best model was selected based on practical significance and statistical indicators (23). Fit indices included Akaike’s Information Criterion (AIC), Bayesian Information Criterion (BIC) and sample size-adjusted BIC (aBIC), with better model fit indicated by lower values. Quality of classification was measured with entropy on a scale of 0-1. Entropy > 0.8 indicated >90% classification accuracy. The bootstrapped likelihood ratio test (BLRT) and the Lo-Mendell-Rubin test (LMR) were used to compare models. At *P*<0.05, the Kth model was better than the K-1 model. Classes were determined using posterior probability. Logistic regression analysis was used to explore socio-demographic and clinical characteristics, coping style, social support and temperament type as predictors of the trajectory of psychological distress. *P*< 0.05 was considered statistically significant.

## Results

### Study population

A total of 155 patients were eligible for this study. 10 patients were lost to follow-up at T2; 8 patients were lost to follow-up at T3, and 5 patients were lost to follow-up at T4. Finally, 132 patients were included in the analysis. Patient socio-demographic and clinical characteristics are summarized in **Table 1**.

**Table 1:** Socio-demographic and clinical characteristics of patients with gynecological cancers.

Characteristic	Value
*Age, mean ± SD (range) years*	55.46 ± 11.12 (28-79)
Place of residence n (%)	
Urban	92 (69.7%)
Rural	40 (30.3%)
*Education level n (%)*	
Junior high school or below	22 (16.7%)
High school/technical secondary school	49 (37.1%),
Junior college	27 (20.4%),
Bachelor's degree or above	34 (25.7%
*Payment method for healthcare n (%)*	
Urban residents' medical insurance	61 (46.3%)
Employees' medical insurance	33 (25%)
New rural cooperative medical system	38 (28.8%)
*Monthly per capita household income n (%)*	
≤1000 Yuan	25 (18.9%),
1000-3000 Yuan	52 (39.4%)
3001-5000 Yuan	36 (27.3%)
≥5001 Yuan	19 (14.4%)
*Occupational status n (%)*	
Employed	43 (32.6%)
Retired	51 (35.6%)
Unemployed	38 (28.8%)
Marital status n (%)	
Unmarried	4 (3%)
Married	121 (91.7%)
Divorced	2 (1.5%),
Widowed	5 (3.8%)
*Childbearing status n (%)*	
No children	9 (6.8%)
Pregnant	123 (93.2%)
*Type of cancer n (%)*	
Cervical cancer	54 (40.9%)
Ovarian cancer	41 (31.1%)
Endometrial cancer	37 (28%)
*Disease Stage n (%)*	
I	17 (12.9%)
II	50 (37.9%)
III	51 (38.7%)
IV	14 (10.6%)
Duration of disease	
< 1 month	60 (45.5%)
1-3 months	50 (37.9%)
> 3 months	22 (16.7%)
Comorbidities	
Yes	47 (35.6%),
No	85 (64.4%)
*Personality dimension (EPRQ)*	
Choleric	35 (26.5%)
Sanguine	32 (24.2%)
Phlegmatic	38 (28.8%)
Melancholic	27 (20.5%)
Psychological distress scores (mean ± SD) *	
T1	6.32 ± 1.51
T2	5.63 ± 1.49
T3	5.07 ± 1.33
T4	4.52 ± 1.50

*ANOVA: F=110.94; P<0.001.

### Psychological distress in patients with gynecological cancers

Among all included (n=132) patients, psychological distress scores showed a statistically significant gradual decline from T1 to T4 (*P*<0.001) (**Table 1**).

**Figure 1:**
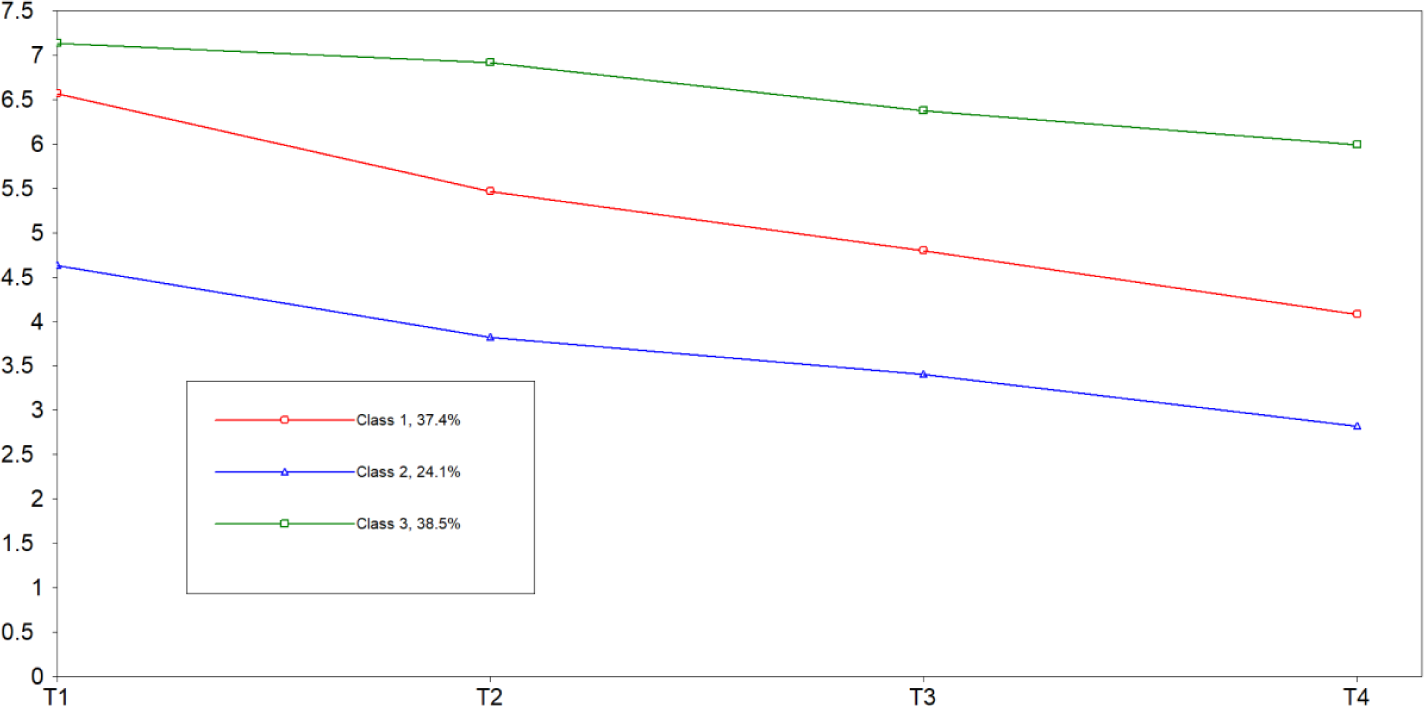
Trajectories of psychological distress in patients with gynecological cancers.

For the LCGM, time was set as a free parameter and 1 to 5 latent classes were extracted. When the number of classes increased from 1 to 2, AIC and aBIC decreased, entropy increased, and the LRT and BLRT were significant (*P*<0.05). When the number of classes increased from 2 to 3, AIC, BIC and aBIC decreased, entropy was highest, and the LRT and BLRT were significant (*P*<0.05). When the number of classes increased from 3 to 4, AIC, BIC, and aBIC decreased, entropy decreased, and the LRT was not significant (*P*>0.05). This suggested that increasing the number of classes was not supported (24). Based on these findings, the theoretical background of psychological distress, and the interpretability of the results, three classes were selected as the final model (**Table 2**). Class 1 included 49 patients (37.4%), Class 2 included 32 patients (24.1%), and Class 3 included 51 patients (38.5%). The trajectories of psychological distress for the three classes are shown in **Figure 1**.

**Table 2:** Model fit.

Category	AIC	BIC	aBIC	Entropy	$\frac{P}{LRT\;\;\;\;\;\;\;\;\;\;\;\;\;\;\;\;\;\;\;\;BLRT}$		Class probability
1	1901.51	1918.81	1899.83	—	—	—	/
2	1668.67	1694.61	1666.15	0.861	0.003	<0.001	0.538/0.462
3	1587.39	1621.99	1584.04	0.873	0.001	<0.001	0.371/0.243/0.386
4	1563.67	1606.92	1559.47	0.822	0.101	<0.001	0.212/0.205/0.303/0.280
5	1477.48	1529.37	1472.43	1.000	0.453	<0.001	0.280/0.144/0.189/0.250/0.136

the “/” in the first row indicates that class probability is not applicable or unavailable for a single-category model, as there is no comparison between different classes.

Each class was named according to its trajectory. According to the cut-off value for psychological distress on the DT recommended by the NCCN, Class 1 (C1 red) showed a high level of psychological distress (*I*=6.06) on the day before surgery (T1), but a subsequent downward trend (*S*>0, *P*<0.05), therefore, Class 1 was named “high level decline “. Class 2 (C2 blue) showed the lowest level of psychological distress at T1 (*I*=4.39), and the level of psychological distress continued to decrease during follow-up at T2-T4 (*S*>0, *P*<0.05). Score on the DT was < 4 points; therefore, Class 2 was named “no psychological distress “. In Class 3 (C3 green), psychological distress on the day before surgery (T1) was the highest (*I*=6.69), and there was no significant change in psychological distress during follow-up (*S*>0, *P*>0.05); therefore, Class 3 was named “high level stable “.

### Single factor analysis of the factors influencing the trajectory of psychological distress in patients with gynecological cancers

Univariate analysis was used to identify factors influencing the trajectory of psychological distress in the 3 classes identified by the LCGM. Patient socio-demographic characteristics, coping style, social support, and temperament type were included in the single factor analysis. Results showed that monthly per capita household income, disease type, coping style, perceived social support, and temperament type were significantly different among the 3 classes (*P*<0.05) (**Table 3**).

**Table 3:** Univariate analysis of factors associated with the trajectory of psychological distress in patients with gynecological cancers.

Item		C1 (n=49)	C2 (n=32)	C3 (n=51)	χ *^2^/F*	*P*	*Back testing*
Monthly per capita household income,n	≤1000	6a	3a	16a	15.124	0.019	
	1001–3000	22a	10a	20a,b			
	3001–5000	16a	10a	10b			
	≥5001	5a	9a	5a,b			
Disease type,n	Cervical cancer	22a	15a	17a	13.029	0.011	
	Ovarian cancer	10a	6a	25b			
	Endometrial cancer	17a	11a	9a			
Temperament type,n	Choleric	11a,b	1a	23a	60.704	<0.001	
	Sanguine	16b	12b	4b			
	Phlegmatic	20b	1a	17a,c			
	Melancholic	2a	18b	7b,c			
Coping style,*(mean ± SD)*	Positive	17.45 ± 2.85a,b	18.50 ± 1.65a	16.84 ± 3.58b	3.125	0.047	C2>C3
	Passive	9.84 ± 1.39a	8.91 ± 1.51b	9.84 ± 2.09c	3.488	0.033	C3>C1>C2
Perceived social support,*(mean ± SD)*		50.39 ± 5.33a,b	51.38 ± 3.96a	48.76 ± 3.83b	3.498	0.033	C2>C3

Different letters (a,b,c) were used to indicate statistical significance (*P*<0.05) while the same letter indicated no statistically significant (*P*≥0.05).

### Logistic regression analysis of factors influencing the trajectory of psychological distress in patients with gynecological cancers

Disordered multinomial logistic regression analysis included psychological distress trajectory as the dependent variable, variables that reached statistical significance in univariate analysis as independent variables, and C1, C2, and C3 assigned values of 1, 2, and 3, respectively. Independent variables were assigned as follows: monthly per capita household income (Yuan), ≤1000 = 1,1001-3000 = 2, 3001-5000 = 3, ≥5001 = 4; disease type: cervical cancer=1, ovarian cancer=2, endometrial cancer=3; disease stage: stage I=1, stage II=2, stage III=3, stage IV=4; temperament type: sanguine=1, choleric=2, phlegmatic=3, melancholic=4.; coping style and social support were entered as original values. Findings showed monthly per capita household income, disease type, positive coping style and temperament type were significant predictors of trajectory of psychological distress in patients with gynecological cancers (*P*<0.05) (**Table 4**).

**Table 4:** Multivariate analysis of factors associated with the trajectory of psychological distress in patients with gynecological cancers.

Dependent variable	Independent variable	B	SE	Wald χ2	P	OR	95%CI
Constant term		-3.901	3.706	1.108	0.292	—	—
C1 vs C3	Disease type						
	Ovarian cancer	-1.357	0.616	4.851	0.028	0.257	0.077-0.861
	Temperament type						
	Sanguine	2.560	1.013	6.388	0.011	12.940	1.777-94.226
Constant term		1.758	5.122	0.118	0.731	—	—
C2 vs C3	Positive coping style Monthly per capita household income	0.276	0.132	4.386	0.036	1.318	1.018-1.707
	1001–3000	-1.933	0.975	3.933	0.047	0.145	0.021–0.978
	Disease type						
	Ovarian cancer	-2.527	1.002	6.363	0.012	0.080	0.011–0.569
	Temperament type						
	Choleric	-5.102	1.387	13.537	<0.001	0.006	<0.001-0.092
	Phlegmatic	-4.463	1.335	11.186	0.001	0.012	0.001–0.158
Constant term		-5.659	5.092	1.235	0.266	—	—
C1 vs C2	Temperament type						
	Sanguine	3.070	0.936	10.757	0.001	21.552	3.440–135.007
	Choleric	5.806	1.507	14.839	<0.001	332.142	17.317–6370.426
	Phlegmatic	6.104	1.444	17.862	<0.001	447.755	26.401–7593.686

The reference group of disease type was endometrial cancer. The reference group of temperament type was depression.

The reference group of monthly per capita household income was ≥5001Y.

Patients with a higher per capita monthly household income were less likely to experience psychological distress. Patients with ovarian cancer were most likely to experience psychological distress. Patients with a positive coping style were more likely to regulate their emotions and have lower levels of psychological distress. Patients with a melancholic temperament type were more likely to experience psychological distress, while patients with a sanguineous temperament type were less likely to experience psychological distress.

## Discussion

### Trajectory of psychological distress in patients with gynecological cancers

This study identified three different trajectories of psychological distress in patients with gynecological cancers based on an LCGM, including Class 1, “high level decline “ (37.4%), Class 2, “no psychological distress” (24.1%), and Class 3, “high level stable” (38.5%). Class 1 and Class 3 accounted for most patients, implying psychological distress is ubiquitous during treatment for gynecological cancers. This is consistent with the findings from a previous longitudinal study in ovarian cancer (19). Patients in Class 1 were in a high level of psychological distress before surgery, which decreased continuously to the end of chemotherapy. We speculate that these patients may adapt to the emotional and physical changes caused by cancer through more positive coping styles. **Table 4** shows that patients in C1 had higher positive coping scores compared with those in C3; a positive coping style may be protective against poor mental health (25). Patients in Class 3 were in a high level of psychological distress before surgery, which remained high to the end of chemotherapy. The patients in this class were more likely to have ovarian cancer, most patients had a melancholic temperament type, lower income, and were unable to regulate emotions well. **Table 4** shows that patients with ovarian cancer were more likely to fall into C3, melancholic patients had a higher probability of being classified into C3, and compared with C2, patients with a per capita monthly income of 1001~3000 had higher psychological distress and probability of being classified as C3. Patients in Class 2 were in a low level of psychological distress throughout their treatment. The level was lower than a longitudinal study on the trajectories of psychological distress in patients with breast cancer (21), possibly because gynecological cancers are associated with poor prognosis and quality of life (22).

### Factors influencing the trajectory of psychological distress in patients with gynecological cancers

#### Disease type

Univariate analysis showed disease type was significantly associated with the trajectory of psychological distress in patients with gynecological cancers in this study. Multivariate logistic regression analysis indicated that patients with ovarian cancer were more likely to be in Class 3 compared to Class 1 or Class 2. Consistent with this, a previous time-dependent study in ovarian cancer survivors showed the pattern of prevalence of mental disorders depended on the nature of disease (26). Ovarian cancer is the leading cause of death among gynecological cancers, with a 5-year survival rate of only 46% (27). Ovarian cancer survivors experience severe long-term fatigue, low sleep quality, and high rates of depression (28). The recurrence rate of ovarian cancer is high (70%-80%) (29), and most patients experience fear of recurrence, resulting in a high level of psychological distress. We recommend that healthcare providers screen for psychological distress in all patients with gynecological cancers, and ovarian cancer in particular, and provide them with timely support and appropriate counseling.

#### Monthly per capita household income

In the present study, univariate and multivariate logistic regression analysis showed patients with lower monthly per capita household income were more likely to be in or Class 3 compared to Class 2. Cancer patients can experience ‘financial toxicity’ related to the cost of care, leading to poor psychosocial health (30, 31). Although China has established a healthcare insurance system, cancer imposes a substantial economic burden on patients and their families, especially for those who are unemployed or have a low income. We recommend that healthcare providers direct patients to social media platforms and charitable organizations to find financial support and possibly reduce psychological distress.

#### Temperament type

Univariate and multivariate logistic regression analysis showed patients with a sanguine temperament in this study were more likely to be in Class 1 compared to Class 2 or Class 3. A sanguine temperament is outgoing with large mood swings, and mood is easily affected by external factors. These patients were in a high level of psychological distress after diagnosis likely because of their lack of knowledge of the disease, and fear of the unknown. These patients rapidly adapted to their new environment and their level of psychological distress decreased during surgical rehabilitation and chemotherapy. Similarly, in a previous study investigating the trajectory of psychological distress from 1 to 2 years after surgery in patients with esophageal cancer, higher dispositional optimism predicted a lower probability of self-reported psychological distress (32).

In the present study, patients with a melancholic temperament were more likely to be in Class 3 compared to Class 1 or Class 2. A melancholic temperament is pessimistic and associated with negative emotions and thoughts. These patients were in a high level of psychological distress before surgery and to the end of chemotherapy. Consistent with this, a previous study showed patients with breast cancer and a melancholic temperament were more likely to experience high-stable psychological distress (33).

It was challenging to assess psychological distress in patients with choleric and phlegmatic temperaments in this study as they do not easily reveal their emotions. We recommend healthcare providers should attempt to understand patients’ temperaments, as this may help guide the need for psychological interventions.

#### Coping style and perceived social support

In the present study, univariate analysis showed positive coping style, passive coping style and perceived social support were significantly associated with the trajectory of psychological distress in patients with gynecological cancers, but the predictive effect of passive coping style and perceived social support was not significant on multivariate logistic regression analysis after controlling for other variables. This may be explained by the small sample size and sample selection bias. Future research is needed to continue to explore the predictive effect of coping style and perceived social support on the trajectory of psychological distress in patients with gynecological cancers.

#### Clinical relevance

Timely and accurate identification and management of patients with cancer who experience psychological distress is essential. Interventions to reduce psychological distress in these patients include psychological/psychosocial therapies and non-pharmacological approaches such as mindfulness, group therapies (talking, cognitive behavioral therapy), creative writing, dignity therapy, web-based apps, life review, problem-solving, couples therapies, physical therapies, and art/music (34).

Psychological distress and the interventions that alleviate it may vary across cancer type and stage of disease. Patients with reproductive, respiratory, upper gastrointestinal, urinary, hematologic, ear/neck/throat, or neuro-oncologic tumors may have the highest risk for psychological distress (35). Brief mindfulness interventions may be most suitable for patients with advanced disease (34). Patients with earlier stage disease could be educated on self-recognition of rising distress and when and where to seek help (36).

Characterizing psychological distress by cancer type will more accurately identify patients with elevated levels of distress and determine the most effective support strategy or intervention (13). This study showed patients with gynecological cancers experience different trajectories of psychological distress, requiring appropriately targeted resources and interventions. Screening for risk factors, including disease type, sociodemographic characteristics and coping styles, before during and after treatment will identify patients at risk of experiencing each trajectory, providing an opportunity to optimize and individualize their care.

#### Limitations

The results of this study should be interpreted in the context of several limitations. This was a single-center study with a small sample size, which may cause sample selection bias and inaccurate conclusions, and only 4 time points during 8 months of follow-up were evaluated. Larger studies conducted at multiple centers with more frequent and longer follow-up are warranted to confirm our findings. Some of the instruments used to measure psychological distress were self-report, which may lead to response bias. Patients with choleric and phlegmatic temperaments are not good at expressing their emotions; therefore, their psychological distress scores may not be accurate. Other variables that might influence psychological distress and its trajectory, such as social support systems and healthcare access, were not fully investigated (37, 38). Large multi-center studies with a longer follow-up and more variables that might influence psychological distress are warranted to comprehensively examine the trajectory of psychological distress in patients with gynecological cancers.

## Conclusions

This longitudinal study used an LCGM to identify three trajectories of psychological distress in patients with gynecological cancers, demonstrating heterogeneity in the trajectory of psychological distress in this patient population. Monthly per capita household income, disease type, positive coping style and temperament type were predictors of the trajectory of psychological distress in the patients in this study. These data provide a reference for future research and should raise awareness among healthcare providers of the need to implement screening measures and timely psychological interventions in patients with gynecological cancers.

## Data Availability

The original contributions presented in the study are included in the article/supplementary material. Further inquiries can be directed to the corresponding author.
